# Activated Clotting Time Monitoring during Atrial Fibrillation Catheter Ablation: Does the Anticoagulant Matter?

**DOI:** 10.3390/jcm9020350

**Published:** 2020-01-27

**Authors:** Anne-Céline Martin, Maeva Kyheng, Vincent Foissaud, Alain Duhamel, Eloi Marijon, Sophie Susen, Anne Godier

**Affiliations:** 1 Hôpital Européen Georges Pompidou, Service de Cardiologie, 20 Rue Leblanc, F-75015 Paris, France; 2 Université de Paris, Innovations Thérapeutiques en Hémostase, INSERM 1140, 4 avenue de l’observatoire F-75006 Paris, France; 3 Université de Lille, CHU Lille, EA 2694 - Santé publique: épidémiologie et qualité des soins, 1, place de Verdun, F-59000 Lille, France; m.kyheng.chr@gmail.com (M.K.); alain.duhamel@univ-lille2.fr (A.D.); 4 Hôpital d’Instruction des Armées Percy, Laboratoire de biologie, 101 avenue Henri Barbusse, F-92140 Clamart, France; vincent.foissaud@intradef.gouv.fr; 5 PARCC-Inserm UMR-S970, Université Paris Descartes, 56 Rue Leblanc, F-75015 Paris, France; 6 Institut d’Hématologie-transfusion, University Lille, CHU Lille, Boulevard du Pr Jules Leclercq, F-59037 Lille CEDEX, France; 7 Fondation Rothschild, Service d’Anesthésie-Réanimation, 29 rue Manin, F-75019 Paris, France

**Keywords:** activated clotting time, direct oral anticoagulants, unfractionated heparin monitoring, atrial fibrillation catheter ablation, overdosing

## Abstract

Atrial fibrillation (AF) catheter ablation is performed in patients receiving direct oral anticoagulants (DOACs) with intra-procedural unfractionated heparin (UFH) administration to achieve activated clotting time (ACT) at 300 s, as for vitamin K antagonist (VKA). We determined whether ACT monitoring might be transposed from VKA to DOAC-treated patients. Blood was taken from 124 patients receiving uninterrupted dabigatran, rivaroxaban, apixaban, or VKA or being untreated. DOAC concentration or INR (VKA) were measured. ACT was determined at baseline, and after spiking with UFH doses equivalent to 1000, 2500, 5000 and 10000 IU in vivo. At baseline, anticoagulants prolonged ACT differently, ACT was longer with dabigatran and shorter with apixaban despite similar concentrations. ACT strongly correlated with INR and dabigatran concentration, but not with apixaban or rivaroxaban concentrations. Moreover, UFH effects on ACT prolongation depended on the anticoagulant: dose-response curves in samples with VKA and dabigatran were parallel whereas ACT prolongation in response to UFH was significantly smaller with rivaroxaban and especially apixaban. Therefore, UFH to achieve ACT at 300 s might be transposed from VKA to uninterrupted dabigatran-treated patients but not to patients receiving FXa-inhibitors, especially apixaban. Targeting 300 s might expose to UFH overdosing and bleeding, questioning the current anticoagulation strategy.

## 1. Introduction

Catheter ablation is increasingly indicated in patients with atrial fibrillation (AF) [1]. This procedure carries a transient but high thromboembolic risk dominated by strokes requiring anticoagulation. This additional anticoagulation leads to an increased risk of major bleeding, including hemopericardium (trans-septal puncture-related or not) and vascular access complications. Guidelines now recommend to perform AF catheter ablation with uninterrupted oral anticoagulation and to administer unfractionated heparin (UFH) intravenously after the trans-septal puncture. To control thrombotic and bleeding risks, and because response to UFH is highly variable, UFH effects must be closely monitored to achieve and maintain an activated clotting time (ACT) equal or greater than 300 s throughout the procedure [2].

For patients receiving vitamin K antagonist (VKA), both UFH monitoring based on the ACT, a validated whole blood coagulation assay, and the ACT target at 300 s are supported by robust evidence [3]. A clear relationship is demonstrated between ACT values, which reflect the level of anticoagulation resulting from both VKA and UFH, and outcomes. A narrow therapeutic window justifies careful monitoring: ACT < 250 s is associated with stroke whereas increasing values beyond ACT ≥ 300 s progressively increases the incidence of bleeding events [4,5]. For patients receiving non-VKA oral anticoagulant (DOAC), anticoagulation management has been extrapolated from VKA experience, without additional specific evidence. Recent open-labelled randomized controlled trials (RCTs) performed in patients with uninterrupted DOAC treatment reported a similar incidence of thromboembolism and bleeding events as for VKA [6,7,8,9]. However, until now, few studies have specifically addressed intra-procedural anticoagulation management. They have all uniformly reported that an unexplained greater amount of UFH was required to achieve a similar ACT target in patients with uninterrupted DOAC, compared to patients receiving VKA [6,8,9,10,11,12]. The management of these procedures cannot safely be resolved without addressing this simple question: can we transpose intra-procedural anticoagulation strategy from VKA to DOACs? To answer this question, we performed an ex-vivo study using samples from patients receiving either DOAC or VKA and untreated-patients. We assessed (1) the relationship between ACT value and the level of anticoagulation, and (2) the effect of spiked fixed UFH doses on ACT prolongation according to the anticoagulant on board. 

## 2. Methods

The ACT Apixaban-Rivaroxaban-Dabigatran (ACTARD) study was an ex vivo study, carried out from June 2016 to February 2018 at Hôpital d’Instruction des Armées Percy, France. The study was approved by the ethical committee (Comité de Protection des Personnes Ile de France V, ref. 15053) and registered at ClinicalTrials.gov (*NCT02839434*). All patients were informed before inclusion and the physician in charge of the patient signed the non-opposition form.

### 2.1. Patients and Study Design

The physician in charge of the study prospectively enrolled patients receiving uninterrupted apixaban, rivaroxaban, or dabigatran at any dose approved for non-valvular AF and who required a blood sampling in their clinical management. Two other groups were considered, namely VKA-treated AF patients with therapeutic INR, and patients free from any anticoagulant treatment. Patients with co-medication such as antiplatelet agents, or parenteral anticoagulants were excluded. 

Blood was collected into haemolysis tubes immediately spiked ex vivo with UFH (heparin calcium Choay^®^ 25 000 UI/5 mL) for a final concentration of 0.2 IU/mL corresponding to 1000 IU UFH intravenous bolus administration in vivo (Figure S1). After 5-minute incubation at room temperature, ACT testing (UFH 0.2-ACT) was performed. Then, UFH was added in previous blood samples to achieve a final UFH concentration of 0.5 IU/mL, corresponding to a 2500 IU UFH bolus administration in vivo and ACT testing (UFH 0.5-ACT) was performed after 5 minute-incubation. Similar protocol was applied for 1.0 and 2.0 IU/mL UFH final concentration, corresponding to 5000 and 10000 IU bolus administration in vivo respectively (UFH 1-ACT and UFH 2-ACT). At the end of the sampling, a drop of blood free from UFH was immediately tested to assess baseline ACT. In addition, blood was collected into 109 mM citrate tube and was centrifuged within 2 h to obtain plasma for coagulation testing.

### 2.2. Coagulation Testing

Coagulation testing included measurement of DOAC concentration for patients receiving apixaban, rivaroxaban and dabigatran or INR for VKA-treated patients. DOAC concentrations were measured using specific commercial assays, dedicated calibrators and controls in accordance with manufacturer recommendations. Quantitative determination of apixaban and rivaroxaban concentrations was assessed with an anti-Xa chromogenic assay, STA-Liquid-anti-Xa^®^ (Stago) and the concentration of dabigatran was measured using the ecarin-based chromogenic assay, STA-ECA II^®^ (Stago). These specific dosages have been validated for the accurate measurement of drugs over a wide range of concentrations up to 500 ng/mL and over 500 ng/mL after plasma dilution. The lower limit of quantification was of 25 ng/mL for apixaban and rivaroxaban measurements and 15 ng/mL for dabigatran measurement. PT was performed according to manufacturer instructions using Neoplastin^®^ CI Plus (Stago) and expressed in ratio (patient PT (s)/normal pool plasma PT (s)) using local normal pool plasma. International Normalized Ratio (INR) was calculated as (PT ratio)^ISI^ using manufacturer-provided generic ISI values (ISI = 1.26).

### 2.3. Activated Clotting Time 

Activated clotting time was measured on whole blood using the point of care Hemochron^®^ Jr. Signature+, with “low range” cartridges (ACT-LR), in accordance with manufacturer’s instructions and performed by a trained physician. Briefly, 15 µL of blood drawn by a 23-gauge catheter were dropped in the cuvette. After mixing with the reagent composed of celite, kaolin, phospholipids, stabilizers and buffer, the sample is moved at a predetermined rate within the test channel and monitored for clot formation by LED optical detectors. When the blood clots, the flow is impeded. This reduction in flow below a predetermined value signals to the instrument that a clot has formed. An internal timer measures the elapsed time between the start of the test and the clot formation, expressed in seconds. The relationship between ACT values and UFH concentrations is linear up to 2.5 IU/mL of blood. The ACT measurement range is 60 to 400 s. If the ACT result is higher than 400 s, an “Out of range-Hi” message is indicated. In these cases, ACT values were coded as equal to 400 s, for the purpose of statistical analysis.

### 2.4. Data Collection

For each patient, age and weight were collected as well as the type, dose and regimen of oral anticoagulation, and the time of the last DOAC intake. Coagulation test results were reported, including apixaban, rivaroxaban or dabigatran concentration for DOAC-treated patients, or INR for VKA-treated patients. Renal function parameters including creatinine level and creatinine clearance according to the Cockcroft–Gault formula were obtained. For all the samples, baseline ACT, UFH 0.2-ACT, UFH 0.5-ACT, UFH 1-ACT, UFH 2-ACT were recorded. ACT value equal or greater than 300 s was selected as the ACT target, in accordance with international guidelines [1,2].

### 2.5. Statistical Analysis 

Continuous variables are expressed as mean (standard deviation, SD). Normality of distribution was assessed using histograms and the Shapiro-Wilk test. In blood samples of patients treated by oral anticoagulants, we evaluated the correlation of ACT with DOAC concentration or INR at baseline by calculating Spearman’s rank correlation coefficients in each oral anticoagulant group (apixaban, rivaroxaban, dabigatran, and VKA). We compared the ACT values assessed at baseline between the 5 study groups (controls, apixaban-, rivaroxaban-, dabigatran, and VKA-treated patients) using one-way analysis of variance (ANOVA); post-hoc pairwise comparisons were adjusted for multiple comparisons by Holm-Bonferroni method. We assessed the effect of increasing UFH doses on ACT values in each study group separately using linear mixed models with a random intercept to account the correlation between samples obtained within the same patients; UFH dose was included as fixed effect. We also used a linear mixed model with a random intercept to compare the ACT changes from samples with UFH to samples without UFH between the 4 oral anticoagulant groups (apixaban, rivaroxaban, dabigatran, and VKA); in this model, treatment group, UFH doses and treatment group*UFH doses interaction were included as fixed effects. Post-hoc comparisons between oral anticoagulant groups at given UFH doses were done using linear contrast after multiple comparison adjustment by Holm-Bonferroni method. Finally, the rate of blood samples with an ACT ≥ 300 s were compared between oral anticoagulant groups for each UFH dose by using the Chi-Square test. Statistical testing was conducted at the two-tailed α-level of 0.05. Data were analyzed using the SAS software version 9.4 (SAS Institute, Cary, NC, USA).

## 3. Results

### 3.1. Baseline Anticoagulation and ACT

#### 3.1.1. Baseline Anticoagulation

**Figure 1:**
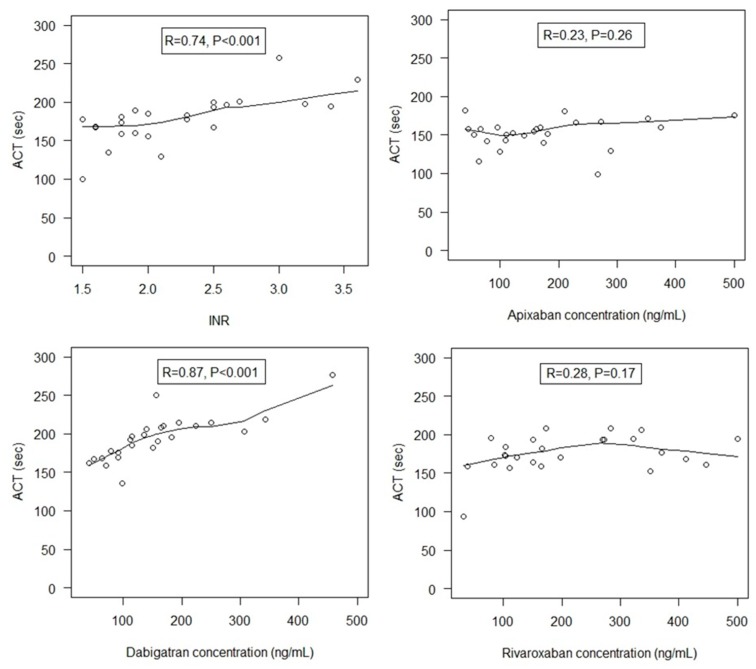
Relationship between activated clotting time (ACT) at baseline and direct oral anticoagulant (DOAC) concentration or International Normalized Ratio (INR). R represents the Spearman’s rank correlation coefficients in each oral anticoagulant group (apixaban, rivaroxaban, dabigatran, and VKA).

Overall, 124 patients were included. One third were female (39/124), mean age was 68 ± 18 years and mean body weight was 79 ± 18 kg. In patients receiving VKA, all INR values were in the recommended range for AF catheter ablation (Table 1). In patients receiving DOACs, there was no difference in DOAC concentrations between groups (*p* = 0.23). A wide inter-individual variability in concentrations was observed though for each DOAC group, with concentrations ranging from 40 to 500 ng/mL, 31 to 500 ng/mL, and 41 to 458 ng/mL, for apixaban, rivaroxaban and dabigatran, respectively (Figure 1). The most frequent time window from the last DOAC dose to blood sampling was 0 to < 4 h, with the same proportion of patients into this time window, in particular in the dabigatran and apixaban groups (Table 2). Mean creatinine clearance was 74 ± 27 mL/min, and no patient had severe renal dysfunction. There was no difference between DOAC groups.

#### 3.1.2. Baseline ACT

At baseline, all oral anticoagulants prolonged the ACT compared to control but in different extent according to the anticoagulant (Table 1): mean ACT were not different in VKA- and rivaroxaban-treated patients, while mean ACT was significantly longer with dabigatran-treated patients compared to all the other anticoagulants, and significantly shorter in apixaban-treated patients compared to all the other anticoagulants. These differences in ACT at baseline were observed despite no difference in DOAC concentrations between the three groups. 

Moreover, the relationship between ACT and the intensity of oral anticoagulation differed according to the anticoagulant on board (Figure 1). ACT strongly correlated with INR (*r* = 0.73, *p* < 0.001) and even more with dabigatran concentration (*r* = 0.87, *p* < 0.0001). By contrast, we did not observe any correlation between ACT and apixaban or rivaroxaban concentrations (*r* = 0.23, *p* = 0.26, and *r* = 0.28, *p* = 0.17, respectively).

### 3.2. Effects of Unfractionated Heparin on ACT

**Figure 2:**
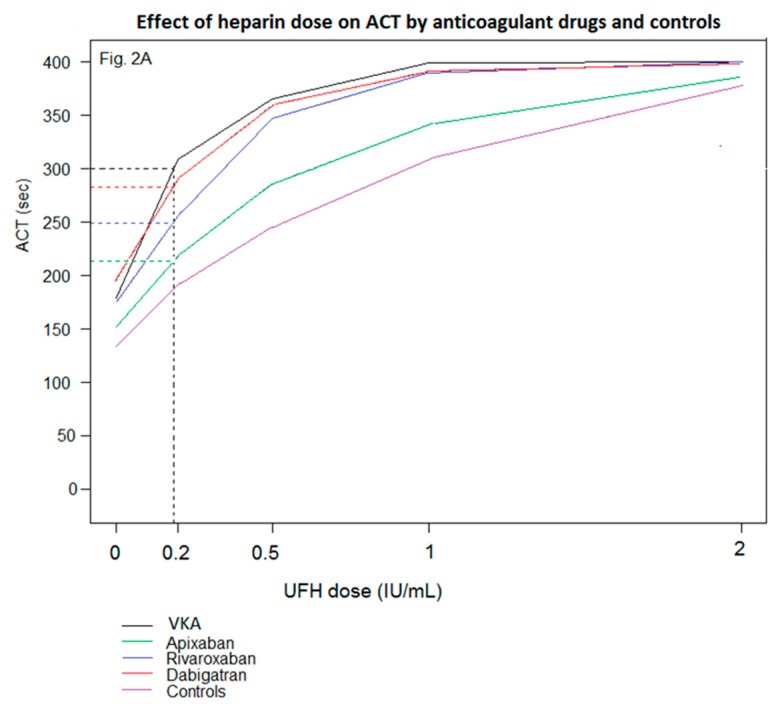

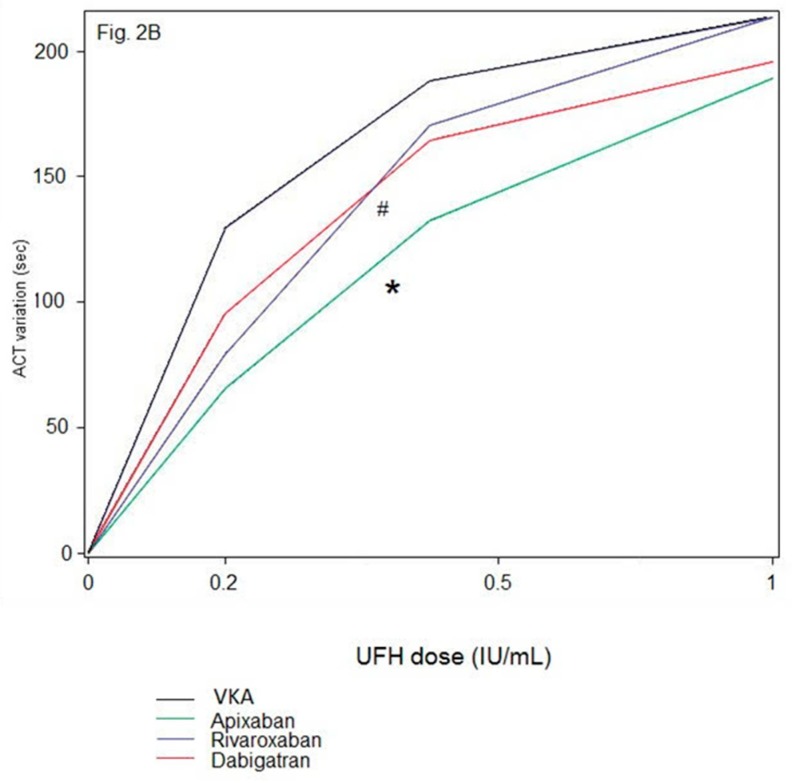
(**A**) Effects of increasing UFH doses on ACT values in patients receiving VKA, apixaban, rivaroxaban, dabigatran et controls. The mean UFH dose required to achieve the ACT target at 300 s in samples from VKA-treated patients (vertical dotted line) lead to an ACT close to 213 s in samples from apixaban treated-patients, an ACT close to 249 in samples from rivaroxaban treated-patients, and an ACT close to 284 in samples from dabigatran treated-patients. (**B**) ACT prolongation in response to increasing UFH doses in patients receiving VKA, apixaban, rivaroxaban, and dabigatran. The ACT dose-response curve to UFH observed in samples from VKA-treated patients was parallel to the curve observed with dabigatran, whereas it differed significantly from the curves observed with rivaroxaban or apixaban between 0 and 0.2 UFH dose (IU/mL) (* *p* < 0.001 for VKA vs apixaban, ^#^ *p* = 0.003 for VKA vs. rivaroxaban).

UFH increased ACT in the five groups, depending on the dose used (Figure 2A). The highest UFH dose induced a prolongation of ACT reaching the upper limit of analytic measurement range (>400 s) in more than 90% of samples thus the results for this dose were excluded for the statistical analysis. 

Incremental doses of UFH prolonged the ACT in different extents according to the oral anticoagulant on board (Figure 2B): the ACT dose-response curve to UFH observed in samples from VKA-treated patients was parallel to the curve observed with dabigatran, whereas it differed significantly from the curves observed with rivaroxaban or apixaban (*p* < 0.001 for VKA vs apixaban, *p* = 0.003 for VKA vs rivaroxaban). Especially, after the first UFH dose, the slopes of the curve were significantly different between VKA and apixaban (*p* < 0.001), as well as between VKA and rivaroxaban (*p* = 0.003).

**Figure 3:**
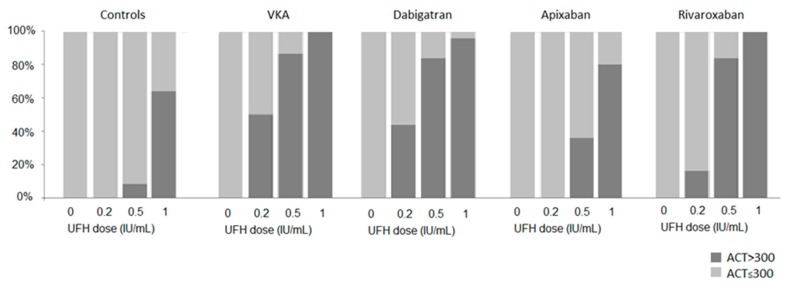
Percentage of patients achieving the ACT target ≥ 300 s in response to each UFH dose, according to the oral anticoagulant on board.

As a result, the proportion of samples achieving the ACT target ≥ 300 s in response to a fixed UFH dose differed significantly according to the oral anticoagulant (*p* < 0.001 for UFH 0.2 IU/mL, *p* < 0.001 for UFH 0.5 IU/mL and *p* = 0.014 for UFH 1 IU/mL) (Figure 3). The ACT response to UFH was highly variable as evidenced by 50% of the samples from VKA-treated patients achieving the ACT target after UFH 0.2 IU/mL. After this UFH dose, only 16% reached ACT ≥ 300 s in rivaroxaban-treated patients and none in apixaban-treated patients. Regarding UFH 0.5-ACT values, 87% and 84% of them were ≥300 s in samples from VKA and dabigatran-treated patients respectively, reflecting an overdosing in half of the samples, as they were already ≥ 300 s after UFH 0.2 IU/mL. A similar UFH dose led to 84% of samples from rivaroxaban-treated patients achieving the ACT target. In contrast, only 36% of them reached the ACT target in apixaban-treated patients and twice the dose of UFH was required for 80% of the UFH 1-ACT values to reach ACT ≥ 300 s. Moreover, Figure 2A suggests that the mean UFH dose required to achieve the ACT target at 300 s in samples from VKA-treated patients led to an ACT close to 213 s in apixaban treated-patients.

## 4. Discussion 

Our study provides new evidence that intra-procedural anticoagulation management for patients undergoing AF catheter ablation with uninterrupted oral anticoagulants cannot be transposed from VKA to all DOAC-treated patients. Our findings demonstrate that ACT monitoring of UFH cannot be applied to patients receiving FXa inhibitors, especially apixaban. Until now, very few studies have addressed the issue of intra-procedural anticoagulation during AF catheter ablation [10,11,12]. They mainly concluded that DOAC-treated patients often require a greater amount of UFH to achieve the ACT target, compared to VKA-treated patients. However, in these studies, pre-procedural DOAC management was heterogeneous, basal ACT was not measured and the effects of a fixed UFH dose were not compared between anticoagulant groups. All these are strong caveats to discover the determinants of intra-procedural UFH differences according to the oral anticoagulant on board. To overcome these limits, we proposed an ex-vivo model analyzing the ACT dose-response to UFH according to the different oral anticoagulants present at baseline. This was achieved using fixed UFH doses, according to a standardized protocol, in plasma samples obtained from patients representative of “real life”, that is, taking VKA or DOAC without interruption prior to AF catheter ablation, resulting in various levels of drug concentrations.

We found that UFH increased ACT values similarly in the presence of VKA and dabigatran, but ACT dose-response to UFH was different between VKA and FXa inhibitors, and between rivaroxaban and apixaban. In other words, a given UFH dose had different effects on the ACT depending on the DOAC on board, whereas intrinsically, at similar antithrombin concentrations (which was the case, data not shown), a given UFH dose has a similar inherent anticoagulant activity in vivo regardless of the oral anticoagulant on board. This result strongly challenges the relevance of ACT target at 300 s as the holy grail and raises the question of the role of the ACT assay as a universal monitoring of intra-procedural UFH in the presence of oral anticoagulants. For patients receiving dabigatran, the similar effects of UFH on ACT prolongation observed when compared to those with VKA, together with the strong correlation between ACT and dabigatran concentrations suggest not only the validity of ACT to monitor UFH but also support the same management of intra-procedural anticoagulation for these patients as for VKA-treated patients, with a similar ACT target ≥ 300 s. In contrast, for patients receiving FXa inhibitors, especially apixaban, ACT strongly underestimates UFH effects, thus leading to overdosing UFH to achieve the ACT target. Moreover, the lack of ACT sensitivity to apixaban, resulting in lower ACT values at baseline, leads to inappropriately higher requirements of UFH, and also contributes to overdosing. 

Consequently, ACT values measured during AF catheter ablation do not reflect the level of anticoagulation resulting from FXa inhibitors and UFH [13]. Running after an ACT target of 300 s is deceptive as it would lead to UFH overdosing compared to VKA-treated patients and inevitably increase the risk of peri-procedural bleeding complications. 

Our results provide the rationale for the differences observed in the recent RCTs. In accordance with our results, in the RE-CIRCUIT trial similar UFH doses and ACT were achieved in dabigatran and VKA-treated patients (12,402 ± 10,721 vs. 11,910 ± 8359 IU, and 330 ± 81 vs. 342 ± 74 s, respectively) [14]. And more UFH was required when the time from the last dabigatran dose to septal puncture increased, since ACT strongly correlates with dabigatran concentration. In contrast, in the VENTURE-AF, the AXAFA-AFNET 5 and more recently, the ELIMINATE-AF trials, a significantly higher dose of UFH was required to achieve ACT > 300 s among rivaroxaban, apixaban and edoxaban-treated patients respectively compared to VKA-treated patients [6,8,9]. Together these results combined with our data, demonstrate that the effect of UFH on ACT prolongation is dramatically altered by the anticoagulant on board. One could argue that more UFH is administered because more UFH is needed. However, this hypothesis is supported neither from a mechanistic point of view nor from consistent available data on the efficacy of FXa inhibitors as compared to VKA in various clinical settings including stroke prevention in AF. Moreover, we provide here the demonstration that UFH doses are increased because UFH (with an intrinsic anticoagulant effect) prolongs the ACT to a lesser extent in the presence of FXa inhibitors compared to dabigatran or VKA, despite a fixe ACT target at 300 s. 

As a result, physicians should be aware that the paradigm validated in patients receiving VKA cannot be applied to those receiving FXa inhibitors, especially apixaban. The development of an alternative comprehensive monitoring device, enabling UFH monitoring in the presence of FXa inhibitors is the way forward. In the meantime, it would be reasonable to change our view on the ACT target and no longer consider ACT ≥ 300 s as an indiscriminately therapeutic target, but rather as a strong signal for supra-therapeutic anticoagulation. Going forward, one option would be to administer a fixed UFH dose corresponding to the average dose given to VKA-treated patients and measure the ACT to check for UFH overdosing rather than to monitor it.

It may be challenging to reconcile our findings with clinical outcomes since none of the open RCTs have sufficient sample size to demonstrate an increase in bleeding complications. In particular, the AXAFA-AFNET 5 trial was underpowered to reliably detect differences in the components of the composite primary outcome, including all-cause death, stroke and major bleeding events [8]. However, the ELIMINATE-AF trial supports our findings about the association between UFH overdosing and bleeding events in the presence of FXa inhibitors [9]. Indeed, the 24% more UFH administered in the edoxaban arm translated into a trend towards higher peri-procedural clinically relevant non-major bleeding events during the ablation and within the first 48 h after compared to the VKA arm. 

## 5. Study limitations

First, UFH doses were selected in our mechanistic model according to their effects on the ACT rather than on the dose itself, to stick closely to clinical practice. Second, this is an ex-vivo study with biological end-points and not with clinical outcomes, and interpretation should be made with caution. However, UFH overdosing must not be ignored and kept in mind since it could increase vascular access complications including haematoma as well as complications related to technical aspects, including hemopericardium, thus would inevitably contribute to difficulties in management. In addition, these results were obtained with a Hemochron^®^ device and may not directly be extrapolated to other point of care devices because of inter-instrumental variabilities. However, given their mechanisms of action we can easily expect similar findings. 

## 6. Conclusion 

Our study provides evidence that for DOAC-treated patients undergoing AF catheter ablation, we should revise the paradigm of the intra-procedural anticoagulation strategy validated in VKA-treated patients, which includes UFH administration to achieve an ACT target at 300 s. While this strategy may be transposed to patients receiving uninterrupted dabigatran, it might not be applied to those receiving uninterrupted FXa inhibitors, especially apixaban. In the presence of FXa inhibitors, targeting ACT at 300 s might expose patients to UFH overdosing and should no longer be considered as an efficacy index but rather as an overdosing signal. These findings question the indiscriminate use of ACT for AF catheter ablation. 

## Figures and Tables

**Table 1:** Comparison of ACT and DOAC concentration between treatment groups.

	INR	DOAC Concentration (ng/mL)	ACT (seconds)
Control (*n* = 25)	-	-	133 (12.0) ^# † ‡ §^
VKA (*n* = 24)	2.2 (0.6)	-	178 (31.9) * ^† §^
Apixaban (*n* = 25)	-	174 (115)	152 (19.3) * ^# ‡ §^
Rivaroxaban (*n* = 25)	-	213 (133)	178 (31.9) * ^† §^
Dabigatran (*n* = 25)	-	158 (98)	195 (29.4) * ^# † ‡^

* *p* < 0.05 when compared to control; ^†^ *p* < 0.05 when compared to apixaban; ^‡^ *p* < 0.05 when compared to rivaroxaban; ^§^ *p* < 0.05 when compared to dabigatran; ^#^ *p* < 0.05 when compared to VKA. Results are expressed in mean (standard deviation). ACT: activated clotting time; DOAC: direct oral anticoagulant; INR: international Normalized Ratio.

**Table 2:** Time from last DOAC dose to blood sampling in each DOAC group.

	Apixaban (*n* = 25)	Rivaroxaban (*n* = 25)	Dabigatran (*n* = 25)
0 to < 4 hours	19	16	19
4 to < 8 hours	6	6	5
≥8 hours	0	3	1

## Supplementary Materials

The following are available online at https://www.mdpi.com/2077-0383/9/2/350/s1, Figure S1: Study design.
