# Supplementary material for: Activated Clotting Time Monitoring during Atrial Fibrillation Catheter Ablation: Does the Anticoagulant Matter?
Source: J Clin Med. 2020 Jan 27;9(2):350. doi: 10.3390/jcm9020350 (PMC7074080; doi:10.3390/jcm9020350)
Supplement: Supplementary file 1 [file jcm-09-00350-s001.pdf]

Activated Clotting Time Monitoring during Atrial Fibrillation Catheter Ablation: Does the Anticoagulant Matter?

Anne-Céline Martin [1,*], Maeva Kyheng [2], Vincent Foissaud [3], Alain Duhamel [2], Eloi Marijon [4], Sophie Susen [5] and Anne Godier[6].

Response to reviewers' comments.

Reviewer #1:

I have read submitted paper "Activated Clotting Time Monitoring during Atrial Fibrillation Catheter Ablation: Does the Anticoagulant Matter" with great attention. This is an interesting study addressing the question of transferring intraprocedural UHF anticoagulation strategy from VKA to non-vitamin K oral anticoagulants. The study design is sound and the submitted manuscript is well written; however, it would still benefit with some slight corrections:

We thank the reviewer for the kind and constructive comments and suggestions, and to give us the opportunity to improve our manuscript.

1. The Authors describe wide inter-individual variability in the measured DOACs concentrations, which may be related with the renal function. Did the Authors assess the renal parameters (creatinine level/eGFR)? I assume that the renal function was normal in all patients - if so it should be stated accordingly in the text.

Inter-individual variability in the response to DOAC treatment expressed by variability in DOAC concentrations has been reported in both healthy volunteers and patients. Many factors may contribute to this inter-individual variability including advanced age, weight and renal dysfunction. On top of these factors, the duration between the last DOAC intake and concentration measurement influences DOAC concentrations.

We assessed age, weight and renal parameters including creatinine level measurement and clearance according Cockcroft and Gault formula: mean age was 68 ± 18 years, mean body weight was 79 ± 18 kg, and mean creatinine clearance was 74 ± 27 mL/min. There was no difference in these parameters between DOAC groups. Moreover, no patient with severe renal dysfunction (creatinine clearance < 30 mL/min) was included.

We modified the "methods" and "results" section of the manuscript:

Page 3 line 123: "For each patient, age and weight were collected as well as the type, dose and regimen of oral anticoagulation, and the time of the last DOAC intake."

Page 3 line 126-127: "Renal function parameters including creatinine level and creatinine clearance according Cockcroft-Gault formula were obtained."

Page 4 line 153-154: "One third were female (39/124), mean age was 68 ± 18 years and mean body weight was 79 ± 18 kg."

Page 4 line 161-162: "Mean creatinine clearance was 74 ± 27 mL/min, and no patient had severe renal dysfunction. There was no difference between DOAC groups."

2. In the lines 91-91 "at the end of the puncture" do the Authors mean the transseptal puncture? It should be clarified.

We thank the reviewer to give us the opportunity to clarify this point.

Baseline ACT measurement needs blood sampled before unfractionated heparin addition. Therefore, we collected a drop of blood from the catheter used for sampling.

"at the end of the puncture" refers to the end of the sampling.

We modified the methods section accordingly (page 3 line 92):

"At the end of the sampling, a drop of blood free from UFH was immediately tested to assess baseline ACT."

3. I would suggest to correct the following passages: „Then considering ACT variations after a same UFH dose, ...” (line 185); „...there is no rational to assume that...” (line 268).

We removed “Then considering ACT variations after a same UFH dose”, page 6 line 190, which is redundant with the following:

“Incremental doses of UFH prolonged the ACT in different extents according to the oral anticoagulant on board (Figure 2B)”

We modified the text accordingly:

Page 9 line 272-274: “However, this hypothesis is supported neither from a mechanistic point of view nor from consistent available data on the efficacy of FXa inhibitors as compared to VKA, in various clinical settings including stroke prevention in AF. “

This minor comments do not affect the great value of your work, which I do appreciate.
